# Acute Smc5/6 depletion reveals its primary role in rDNA replication by restraining recombination at fork pausing sites

**DOI:** 10.1371/journal.pgen.1007129

**Published:** 2018-01-23

**Authors:** Xiao P. Peng, Shelly Lim, Shibai Li, Lisette Marjavaara, Andrei Chabes, Xiaolan Zhao

**Affiliations:** 1 Molecular Biology Program, Memorial Sloan Kettering Cancer Center, New York, NY, United States of America; 2 Tri-Institutional MD-PhD Program of Weill Cornell Medical School, Rockefeller University, and Sloan-Kettering Cancer Center, New York, NY, United States of America; 3 Department of Medical Biochemistry and Biophysics, Umeå University, Umeå, Sweden; Columbia University, UNITED STATES

## Abstract

Smc5/6, a member of the conserved SMC family of complexes, is essential for growth in most organisms. Its exact functions in a mitotic cell cycle are controversial, as chronic Smc5/6 loss-of-function alleles produce varying phenotypes. To circumvent this issue, we acutely depleted Smc5/6 in budding yeast and determined the first cell cycle consequences of Smc5/6 removal. We found a striking primary defect in replication of the ribosomal DNA (rDNA) array. Each rDNA repeat contains a programmed replication fork barrier (RFB) established by the Fob1 protein. Fob1 removal improves rDNA replication in Smc5/6 depleted cells, implicating Smc5/6 in the management of programmed fork pausing. A similar improvement is achieved by removing the DNA helicase Mph1 whose recombinogenic activity can be inhibited by Smc5/6 under DNA damage conditions. DNA 2D gel analyses further show that Smc5/6 loss increases recombination structures at RFB regions; moreover, *mph1∆* and *fob1∆* similarly reduce this accumulation. These findings point to an important mitotic role for Smc5/6 in restraining recombination events when protein barriers in rDNA stall replication forks. As rDNA maintenance influences multiple essential cellular processes, Smc5/6 likely links rDNA stability to overall mitotic growth.

## Author summary

Smc5/6 belongs to the SMC (Structural Maintenance of Chromosomes) family of protein complexes, all of which are highly conserved and critical for genome maintenance. To address the roles of Smc5/6 during growth, we rapidly depleted its subunits in yeast and found the main acute effect to be defective ribosomal DNA (rDNA) duplication. The rDNA contains hundreds of sites that can pause replication forks; these must be carefully managed for cells to finish replication. We found that reducing fork pausing improved rDNA replication in cells without Smc5/6. Further analysis suggested that Smc5/6 prevents the DNA helicase Mph1 from turning paused forks into recombination structures, which cannot be processed without Smc5/6. Our findings thus revealed a key role for Smc5/6 in managing endogenous replication fork pausing. As rDNA and its associated nucleolar structure are critical for overall genome maintenance and other cellular processes, rDNA regulation by Smc5/6 would be expected to have multilayered effects on cell physiology and growth.

## Introduction

The conserved Smc5/6 complex (or Smc5/6) is required during normal growth and for coping with genotoxins [1–4]. Due to the essential nature of the complex, studies thus far have examined partial loss of function mutants of the complex in various organisms. As its chronically sick alleles give varied phenotypes, a coherent view of Smc5/6 function during growth has yet to be established. In budding yeast, studies using temperature sensitive alleles suggest that Smc5/6 is required during S phase, as shifting to non-permissive temperatures during S, but not G2-M phase, leads to defects [5]. However, two views about the S phase functions of Smc5/6 have been proposed based on distinct mutant phenotypes. One *smc6* allele (*smc6-56*) impairs replication of longer chromosomes while another (*smc6-9*) only diminishes the duplication of chromosome XII (Chr XII), which harbors the entire ribosomal DNA (rDNA) array [5–7]. The former defect was interpreted as reflecting Smc5/6 roles in replication fork rotation [6], while the mechanism for the latter defect was unclear [5,7]. More recently, another study proposed that Smc5/6 is essential in G2, but not S phase, as fusion of Smc5/6 with a G2-cyclin, but not S-cyclin, cassette sustained cell growth [8]. A number of factors likely contribute to the diverse phenotypes observed for this collection of chronic alleles. For example, cells containing chronically altered alleles can accumulate different levels or types of stress over generations, engendering a phenotype compounded from primary defects and various secondary changes. Indeed, *smc5/6* alleles show alterations in diverse processes, including cell cycle checkpoint responses, cohesin function, repair pathway usage, and centromeric regulation [9–12]. As such, it is a challenge to deconvolute chronic mutant phenotypes and derive primary roles for Smc5/6.

The lack of a cohesive understanding of primary Smc5/6 defects in normal cell growth hinders advances in the field and is an important issue to address. An effective way to overcome the drawbacks of chronic allele usage is to induce acute and potent Smc5/6 depletion, which enables identification of the immediate consequences of Smc5/6 loss. However, robust loss of Smc5/6 within one cell cycle is difficult to achieve. For example, the budding yeast Smc5/6 subunits appear to be stable and even low levels of the complex can be tolerated for multiple cell divisions [13]. Previous strategies using conditional promoters or DHFR-degron systems reduced Smc5/6 protein levels and cell growth, but failed to cause the null phenotype of lethality [14,15]. To improve the robustness of Smc5/6 depletion, we turned to an auxin-inducible degron (AID) system [16]. We found that while targeting a single subunit of the Smc5/6 complex with AID did not cause lethality, a null phenotype could be achieved by combining AID fusions of two subunits. Thus, we used this Smc5/6 ‘double degron’ system to examine the immediate consequences of robust Smc5/6 degradation in the first cell cycle after loss. Our findings using this system demonstrate that the primary effect of Smc5/6 loss is defective rDNA replication in yeast. We present further genetic and DNA analysis data to derive a mechanism by which Smc5/6 promotes rDNA replication. Our data suggest that Smc5/6 is involved in managing programmed replication pausing at rDNA and restrains Mph1-mediated recombinogenic events to enable proper duplication of this at-risk locus.

## Results

### Smc5/6 degron design and growth assessment

To circumvent the limitations of *smc5/6* hypomorphs used in previous studies, we employed an inducible, plant hormone-based, AID degron system to achieve acute depletion of Smc5/6 complex subunits. This system exploits the ability of a diffusible plant hormone auxin (IAA) to bridge the interaction between a plant adapter protein (TIR1)-bound endogenous ubiquitin ligase complex (SCF) and a TIR1-binding cassette (AID) fused to a target protein [16]. IAA addition recruits AID-fusion proteins to the TIR1-SCF ubiquitin ligase complex, which polyubiquitinates them for proteasome-mediated degradation (S1A Fig). Neither TIR1 nor functional concentrations of IAA are toxic to cells or interfere with other cellular processes, and rapidly inducible degradation has been reported for many yeast proteins using this system [17].

**Fig 1:**
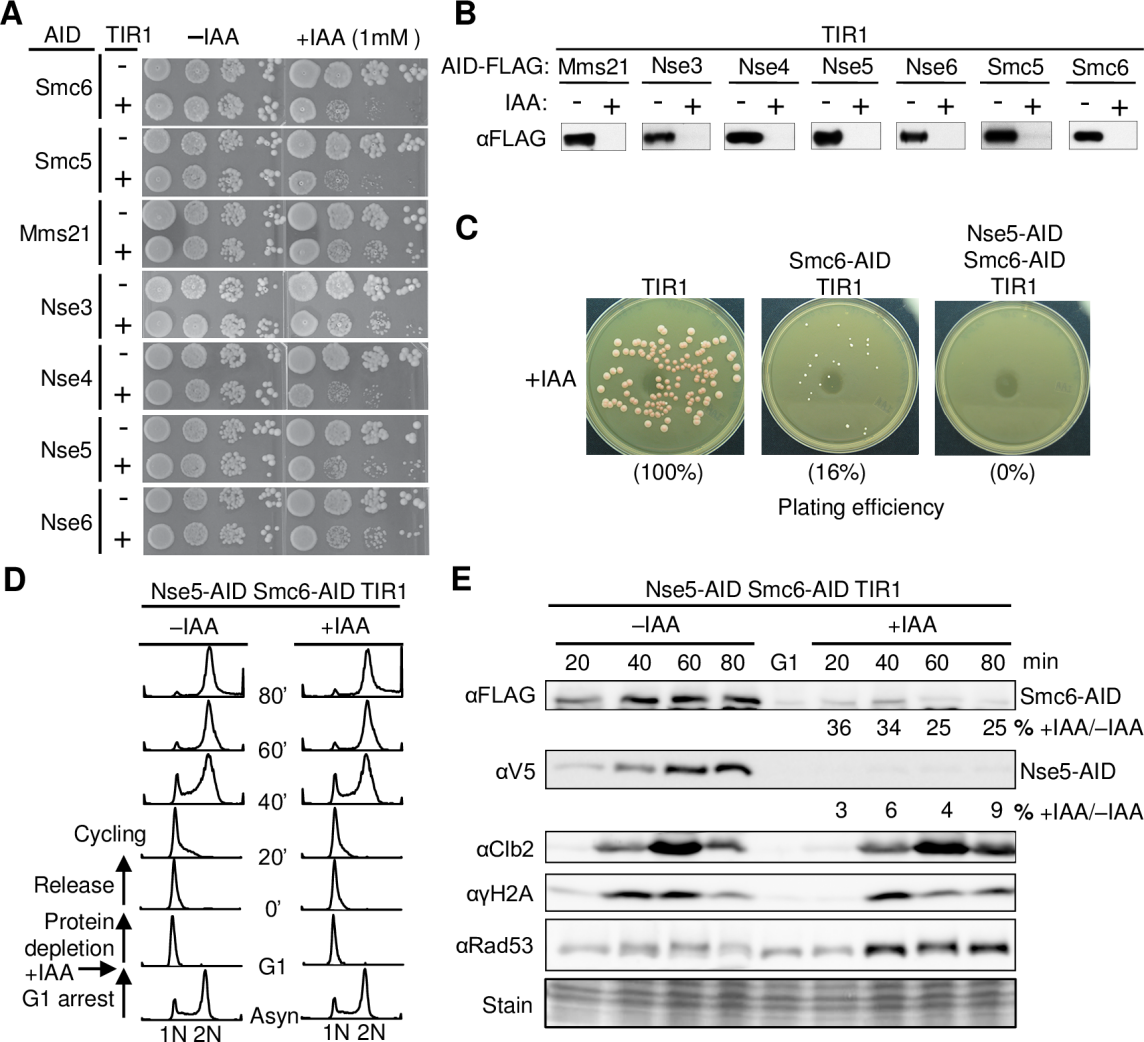
Smc5/6 loss of function is enhanced by combining degrons. A. Growth assessment of strains containing AID-tagged Smc5/6 subunits with or without TIR. Ten-fold serial dilutions of log phase cells were spotted onto medium with or without IAA. Tagging each of the seven indicated subunits slows cell growth to different degrees only in the presence of IAA and TIR1. B. Examination of protein degradation in degron strains by Western blot. IAA-treated (90 min) and un-treated asynchronous cultures were examined. All AID subunits tested achieved protein loss. C. Combining Nse5 and Smc6 degron alleles results in greater growth defects than the Smc6 degron alone can achieve. The same number of cells for each indicated strain was plated on media containing 1 mM IAA and grown at 30°C for 2 days. The percentages indicate the average number of colonies formed in each strain relative to TIR1-only controls. D. Experimental scheme and cell cycle progression of Nse5-Smc6 double degron cells. For +IAA samples, cells were arrested in G1 phase by alpha factor, treated with 1 mM IAA for 90 min, released from G1 arrest into fresh IAA-containing media and allowed to progress through the first cell cycle under protein depletion conditions as assayed by FACS analyses. The procedure for–IAA samples was the same, except no IAA was added. E. The Nse5-Smc6 double degron strain was examined for protein degradation and markers of cell cycle progression and DNA damage. Western blots were performed on samples harvested from the time course experiments (D). Protein levels in IAA-treated cells are expressed as a relative percentage of those for untreated cells at each time point after G1 release. Molecular markers of cell cycle progression (Clb2) and DNA damage (Rad53 and γH2A) were examined. Note that both Nse5 and Smc6 protein levels are lower in G1 than in S phase for untreated cells.

Each of the eight subunits of the Smc5/6 complex (Smc5, Smc6, Mms21, and Nse1, 3–6) was tagged with a C-terminal AID at its endogenous locus. Without IAA or TIR1, these strains gave rise to wild-type sized colonies, except for the Nse1-AID allele, which showed slow growth and was thus excluded from further analyses (Fig 1A). IAA addition elicited a slow growth phenotype in strains containing AID-tagged Smc5/6 alleles only if TIR1 was also present (Fig 1A). Immunoblotting for targeted proteins confirmed significantly reduced levels within 90 minutes after IAA addition (Fig 1B). These results indicate that single Smc5/6 AID degron alleles cause growth defects.

As Smc5/6 null alleles are lethal [2], the slow growth seen with single AID degrons of Smc5/6 subunits suggested that cells tolerate low levels of Smc5/6. Similar conclusions were reached in previous studies titrating cellular levels of Smc5/6, or another SMC complex, cohesin [14,15,18]. To enhance the robustness of Smc5/6 depletion, we constructed combined degrons based on the following rationale. We observed that loss of one subunit of the yeast Smc5/6 complex generally did not affect the levels of the other obligate members of the complex (S1B–S1D Fig). Thus, reducing the level of a second subunit should further decrease the probability of forming intact complexes. Indeed, we found that combining AID degrons of Nse5 and Smc6 proved highly effective for eliminating colony formation upon IAA treatment (Fig 1C). Thus, Nse5-AID Smc6-AID strains containing TIR1, referred to as “Nse5-Smc6 double degron” or “double degron” for simplicity, were used to investigate the primary defects caused by Smc5/6 complex depletion.

### Degradation efficiency and cell cycle profiles of the Nse5-Smc6 double degron strains

To evaluate whether the Nse5-Smc6 double degron strain suffers chronic defects prior to induced protein degradation, we compared it to three frequently used hypomorphic alleles: *smc6-56*, *smc6–P4*, and *mms21-11* [2,6,19]. These three mutants grow more slowly than wild-type cells at permissive temperatures and are lethal at 37°C [2,6,19]. Even at permissive temperatures, they showed higher levels of dNTPs, a condition associated with increased DNA stress and altered replication profiles (S2A Fig) [20,21]. In contrast, cells harboring the Nse5-Smc6 double degron exhibited dNTP levels similar to wild-type or strains containing TIR1 alone, in both G1 and asynchronous cultures (S2B Fig). These data suggest that Nse5-Smc6 double degron cells lack chronic genome stress prior to induced degradation with IAA. The double degron strain’s normal growth and wild-type dNTP levels under un-induced conditions offer a more optimal baseline for detecting primary defects after Smc5/6 depletion.

We also examined the extent of Nse5 and Smc6 protein loss upon IAA addition and effects on bulk DNA replication. To this end, G1-arrested double degron strains were treated with IAA for 90 min to induce protein degradation, and then released into cycling with IAA (Fig 1D, +IAA). As a control, the same strains were examined in parallel without IAA (Fig 1D,–IAA). Protein levels were monitored at four time points after release from G1 (Fig 1E). Relative to controls, double degron cells in IAA lost ~95% of Nse5 and 70% of Smc6 proteins in the first cell cycle (20–80 min) (Fig 1E). It is reasonable to infer that levels of the intact complex are likely lower than 5% of those in wild-type cells, as depletion of each subunit independently affects the complex.

Next, we assessed bulk genome replication by FACS analysis. Throughout the time course, the double degron cells showed similar cell cycle progression in the presence or absence of IAA (Fig 1D). On a molecular level, Clb2 kinetics, an indicator of cell cycle progression [22], were also comparable (Fig 1E). IAA-treated double degron cells also showed no increased phosphorylation of the Rad53 checkpoint kinase or gross differences in levels of γH2A, a marker for DNA replication or breaks (Fig 1E) [23,24]. Our data suggest that there are no detectable changes in cell cycle progression, DNA break marker increase, or checkpoint activity in the first cell cycle upon Smc5/6 subunit removal. Moreover, cells appear to undergo normal bulk genome replication even with robust Smc5/6 depletion.

### Nse5-Smc6 double degron cells show first cell cycle replication defect specific to Chr XII

**Fig 2:**
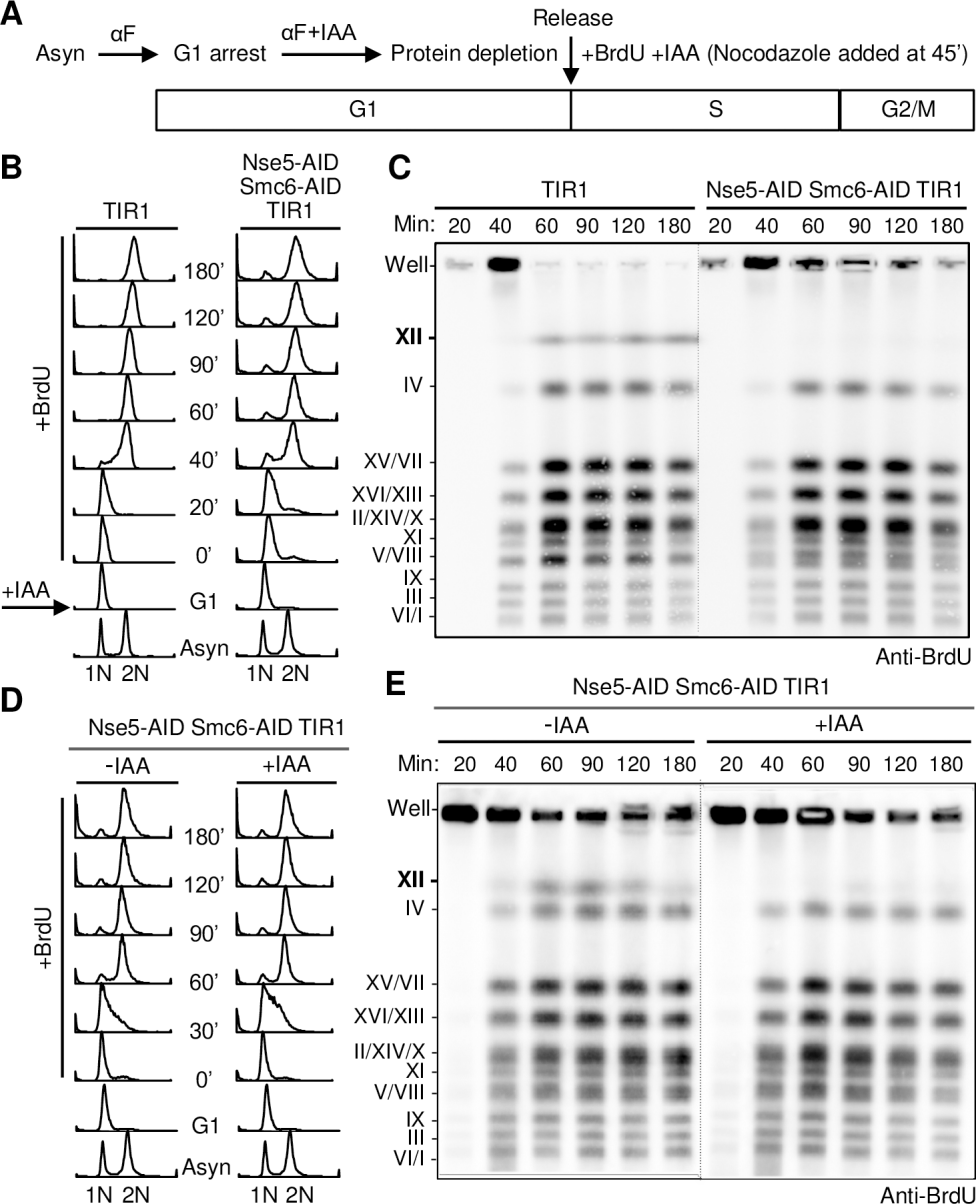
Chr XII replication is affected in the first cell cycle after Smc5/6 depletion. A. Experimental scheme of alpha factor (aF)-induced G1 arrest followed by release into cycling for examination of chromosomal replication. This procedure was used for subsequent figure panels unless otherwise noted. B. FACS profiles of IAA-treated Nse5-Smc6 double degron and control (TIR alone) cells after G1 arrest and synchronized release into first cell cycle progression. C. Western blot showing reduced BrdU incorporation for Chr XII in degron versus control cells in the first cell cycle. Chromosomes were separated by PFGE and new DNA synthesis was detected using an anti-BrdU antibody. D. FACS profiles of G1 arrest and release assays using Nse5-Smc6 double degron treated or not treated with 1 mM IAA. E. Western blot showing reduced Chr XII BrdU incorporation in IAA-treated cells compared with untreated cells in the first cell cycle after G1 release. New DNA synthesis in each chromosome was detected using an anti-BrdU antibody after PFGE. Samples in D and E were run on the same gel, with dotted lines indicating the junction that separates samples from the two strains. The same labeling convention is used for subsequent figures showing PFGE data.

To achieve more sensitive detection of chromosome synthesis in the first cell cycle of Smc5/6 loss than that afforded by FACS, we used BrdU incorporation coupled to pulsed field gel electrophoresis (PFGE). PFGE can separate replicated chromosomes, which enter the gel, from the branched forms still undergoing replication, which remain trapped in the wells [25]. BrdU labels the newly synthesized DNA in each replicated chromosome and can be detected by immunoblotting [26]. To apply these techniques, we used a similar procedure as described above and monitored cells for 180 min after G1 release (Fig 2A). To capture new DNA synthesis, we added BrdU immediately after G1 release. We also used nocodazole to prevent additional rounds of cycling in order to focus on effects of Smc5/6 depletion in the first cell cycle (Fig 2A).

Nse5-Smc6 double degron cells were first compared to cells containing the TIR1 adaptor but without degron alleles. As before (Fig 1D), FACS data showed that both strains were synchronized in G1, progressed through S phase, and achieved G2/M arrest (Fig 2B). For control cells, BrdU signals for all chromosomes increased from 20’ to 40’ and peaked at 60’ (Fig 2C). This is consistent with the FACS profile, which shows bulk replication having largely completed by 60’ (late S phase), with cells remaining in G2/M for subsequent time points. Strikingly, both BrdU blotting and DNA staining of PFGE gels showed little to no replicated Chr XII signal in Nse5-Smc6 double degron cells throughout the duration of the time course, despite wild-type-like cell cycle progression (Fig 2C; S3A Fig). Quantification of BrdU signals for other chromosomes found no significant differences between IAA-treated double degron and control cells (S3B Fig).

To verify that the lack of fully replicated Chr XII signal was due to the loss of AID-targeted proteins, we repeated the assay with double degron strains in the presence or absence of IAA. Identical first cell cycle progression was seen for both conditions (Fig 2D). Once again, only IAA-treated degron cells showed low Chr XII signal, as detected by both BrdU incorporation and DNA staining (Fig 2E; S3C Fig). Based on these results, we concluded that acute Smc5/6 depletion leads to a Chr XII-specific replication defect in the first cell cycle.

### Eliminating Fob1 improves Chr XII replication in Nse5-Smc6 double degron cells

**Fig 3:**
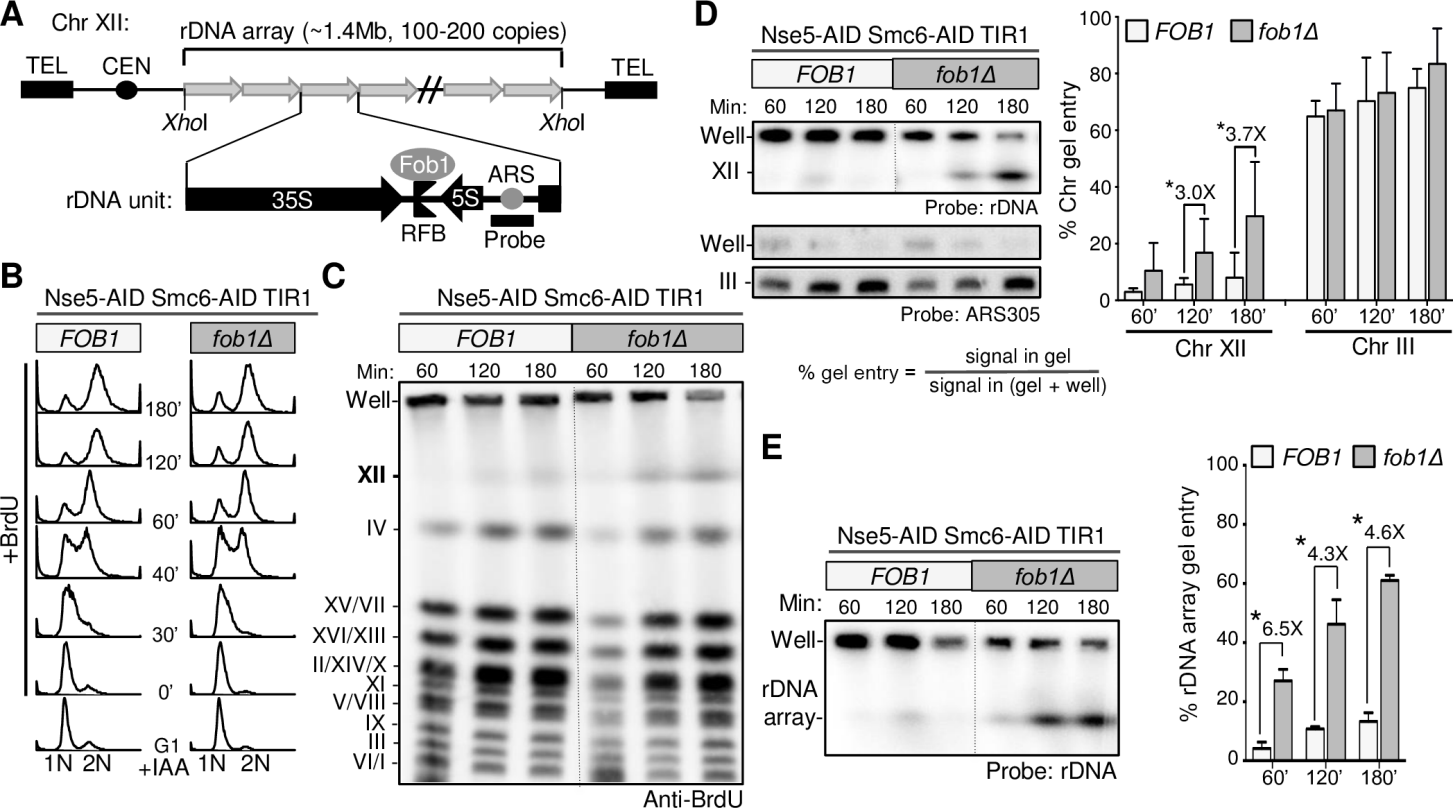
Chr XII replication defect in Nse5-Smc6 double degron cells is improved by Fob1 removal. A. Diagram showing the rDNA array on Chr XII and key features of each rDNA repeat. Fob1 binds to the RFB in each rDNA repeat to block replication forks moving opposite the direction of 35S rDNA transcription. Replication of an rDNA repeat begins at the rARS replication origin. Note that XhoI digests an intact rDNA array out of Chr XII. The probe used for detecting Chr XII and rDNA on Southern blots is depicted. B. FACS profiles showing Nse5-Smc6 double degron (*FOB1*) and Nse5-Smc6 double degron *fob1∆* (*fob1∆*) cells progressing through the first cell cycle of a G1-arrest and release assay as described in Fig 2A. C. Western blotting with anti-BrdU antibody of PFGE-separated chromosomes shows that *fob1∆* increases in-gel Chr XII BrdU signals in degron cells progressing through the first cell cycle. D. Southern blots for Chr XII and Chr III showing that the *fob1∆* effect is Chr XII-specific. Chr XII and Chr III signals in-gel and in wells were detected using radiolabeled probes against rDNA or the ARS305 region of Chr III, respectively. Percentage of chromosome gel entry was calculated as described. Standard deviations and P-values (t-test, *p<0.05) are derived from n = 6 trials for rDNA and n = 3 trials for ARS305. E. *fob1∆* reduces the rDNA replication defect in Nse5-Smc6 double degron cells. Chromosomes were digested with XhoI before PFGE and then the rDNA array was detected by Southern blotting with an rDNA-specific probe. Percentage of rDNA gel entry was calculated as described in (D). Standard deviations and P-values (t-test, *p<0.05) are derived from n = 3 trials for rDNA.

Chr XII is unique among yeast chromosomes in that it harbors the entire rDNA array. This large array (~1.4 Mb) represents 10% of the yeast genome and half of Chr XII, and is intrinsically difficult to replicate. Uniquely, each of the 100–200 rDNA repeats in the array contains a programmed *r*eplication *f*ork *b*arrier, or RFB, located between the 5S and 35S rRNA genes (Fig 3A) [27]. When bound by Fob1, the RFB sequence can block replication fork progression in the direction of 35S rRNA transcription [28]. This mechanism helps avert collisions between the replication and transcription machineries, but also requires careful management to enable replication completion and avoid repeat instability [29].

Knowing that rDNA harbors these specific sites of replication blockade, we asked whether their removal could ameliorate the observed Chr XII replication defect of Smc5/6-depleted cells. To this end, we removed Fob1 in the Nse5-Smc6 double degron strains, and repeated the BrdU and PFGE tests described above (Fig 2A). Both degron and degron *fob1∆* strains showed identical first cell cycle FACS profiles (Fig 3B). Importantly, the fully replicated Chr XII BrdU signal was stronger in degron *fob1∆* strains than in degron strains at 120’ and 180’ after release from G1 (Fig 3C). This finding was further confirmed by Southern blotting with an rDNA-specific probe (Fig 3D). Quantification showed ~3-fold increased Chr XII signals in degron *fob1∆* cells over degron alone (Fig 3D). As expected, these *fob1* effects were specific to Chr XII, as signals for other chromosomes such as Chr III were similar for the two strains (Fig 3D).

### *fob1∆* improves rDNA replication in Nse5-Smc6 double degron cells

To determine if the observed *fob1∆* effect on Chr XII replication in double degron cells reflects an improvement of rDNA replication per se, we directly examined the rDNA array, which can be released from Chr XII by the restriction enzyme XhoI. The rDNA array is flanked by XhoI recognition sites but contains no internal ones, so XhoI cleavage releases the entire array from its chromosomal context [30]. The array’s large size enables its resolution from other smaller chromosome fragments by PFGE and can be subsequently detected by hybridization to an rDNA-specific probe. As shown in Fig 3E, ~90% of the rDNA signal failed to enter the gel in degron cells even by 180 min, consistent with our data for intact Chr XII (Fig 3D). This confirmed that the rDNA array itself suffers from incomplete replication when Smc5/6 is depleted. The rDNA of degron *fob1*Δ cells entered the gel from 60 to 180 minutes, and in-gel levels of rDNA were 4–6 fold greater than those of degron cells (Fig 3E). The ability of *fob1Δ* to improve replication of the rDNA array more than that of Chr XII (Fig 3D and 3E) confirms that rDNA is responsible for the beneficial effect exerted by *fob1Δ* on Chr XII replication in double degron cells.

We also asked whether replication fork blockade by Fob1-RFB, outside the context of rDNA, were sufficient to create a requirement for Smc5/6. It is known that two RFB sites inserted on Chr III can pause replication forks upon Fob1 overexpression, and that such pauses are resolved by the recruitment of the Rrm3 helicase [31]. We found that Smc5/6 loss in this system impaired Chr XII replication as expected, but did not affect Chr III replication (S4 Fig). Thus, additional properties specific to the rDNA locus not recapitulated by these RFB sites contribute to the importance of Smc5/6 for rDNA replication (see Discussion).

### Eliminating Mph1 improves Chr XII and rDNA replication in Nse5-Smc6 double degron cells

We then investigated which other protein factors might play a role in Smc5/6-dependent effects at rDNA. Previous studies showed that Smc5/6 inhibits the pro-recombinogenic activity of the Mph1 DNA helicase at stalled forks under DNA damage conditions [19,32–34]. Although Mph1 has not been implicated in rDNA and Fob1-RFB-mediated replication pausing, replication forks stalled by Fob1-RFB, like those stalled by template lesions, require management to ensure replication completion. Thus, we tested whether Smc5/6 inhibition of Mph1 may also be relevant at endogenous rDNA fork blockage sites.

**Fig 4:**
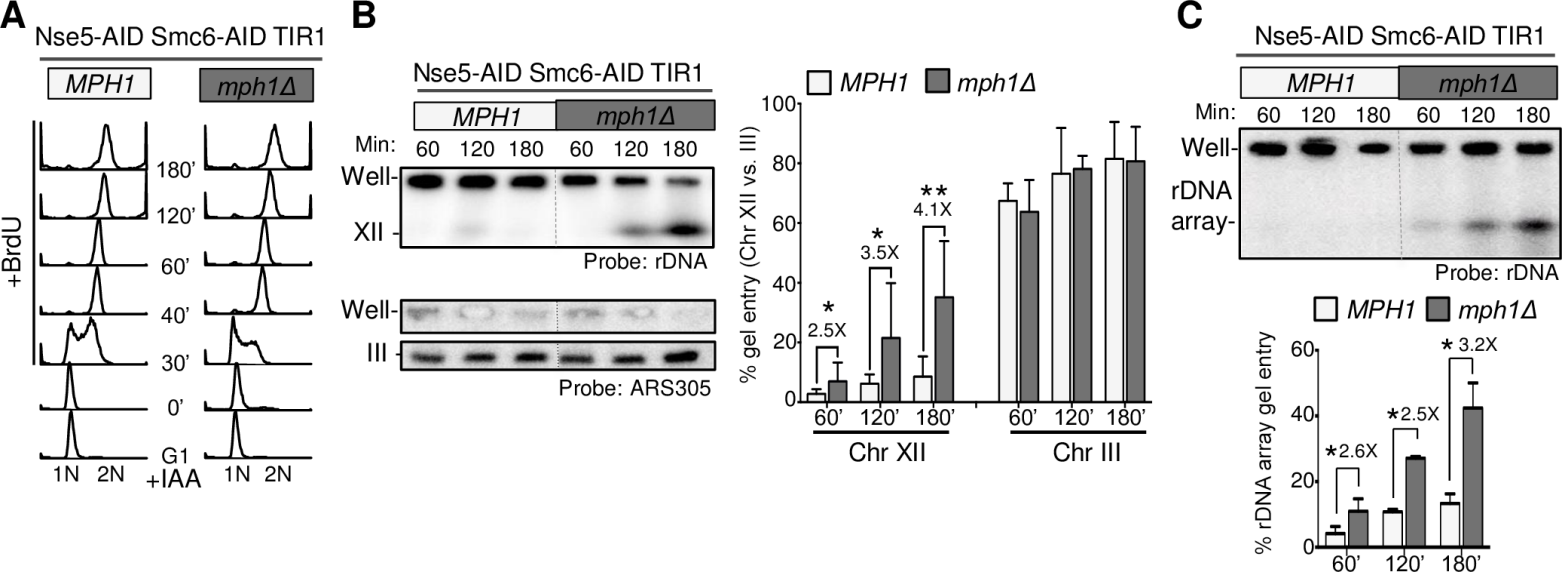
*mph1Δ* reduces rDNA replication defects in Nse5-Smc6 degron cells. A. FACS profile showing first cell cycle progression of Nse5-Smc6 double degron (*MPH1*) and Nse5-Smc6 double degron *mph1Δ* (*mph1Δ*) cells using the same strategy shown in Fig 2A. B. Southern blots for Chr XII and Chr III showing that *mph1Δ* improves Chr XII replication. Chr XII and Chr III signals in wells and in-gel were detected, and percentage of chromosome gel entry was calculated as described in Fig 3D. Standard deviations and P-values (t-test, *p<0.05, ** p<0.01) are derived from n = 3 trials for rDNA and n = 3 trials for ARS305. C. *mph1Δ* reduces rDNA replication defect in Nse5-Smc6 double degron cells. Chromosomes were digested with XhoI before PFGE and Southern blot as in Fig 3E. Percentage of rDNA gel entry was calculated in Fig 3E. Standard deviations and P-values (t-test, *p<0.05) are derived from n = 3 trials.

To this end, we deleted *MPH1* in Nse5-Smc6 double degron cells. FACS profiles of both degron and degron *mph1Δ* showed identical first cell cycles (Fig 4A). Using the procedure described above, we found that *mph1Δ* increased levels of fully replicated Chr XII by about three-fold in degron cells (Fig 4B). This effect was similar to *fob1Δ*, although significant suppression was seen by 60’ for *mph1Δ*, but not *fob1Δ*. Furthermore, when examining the rDNA array within Chr XII by XhoI digestion, we found that its duplication in degron *mph1Δ* cells increased to levels similar to that of Chr XII (Fig 4C). We note an overall trend of weaker suppression of rDNA replication than Chr XII as a whole by *mph1Δ*, while *fob1Δ* had the opposite trend. This would be consistent with a role for Mph1, but not Fob1, at Chr XII loci outside rDNA.

### Fob1 and Mph1 removal are epistatic for improving replication in Nse5-Smc6 double degron cells

**Fig 5:**
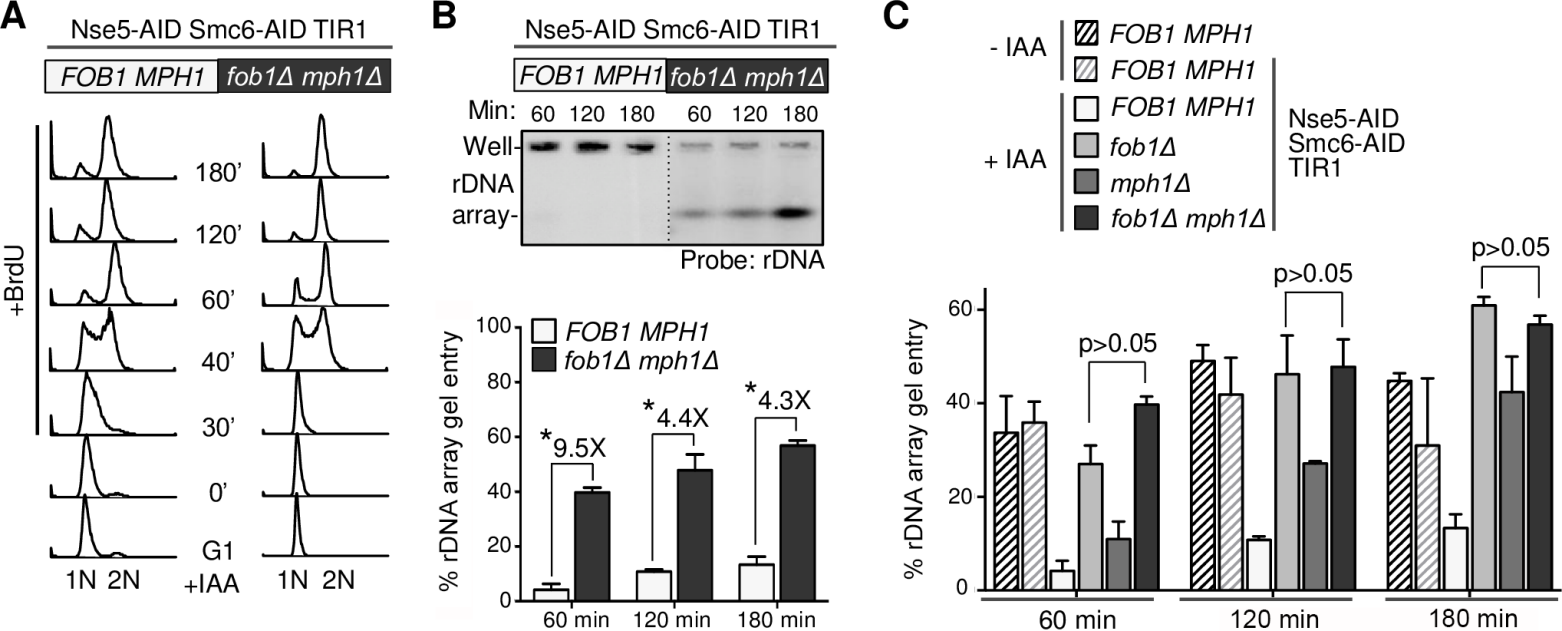
*fob1Δ mph1Δ* improves rDNA replication in Nse5-Smc6 degron cells. A. FACS profile showing first cell cycle progression of Nse5-Smc6 double degron (*FOB1 MPH1*) and Nse5-Smc6 double degron *fob1Δ mph1Δ* (*fob1Δ mph1Δ*) cells. Experiments followed the scheme shown in Fig 2A. B. XhoI-digested samples were subjected to PFGE and Southern blot to examine rDNA array replication. Signals from an rDNA-specific probe are quantified as in Fig 3E. Standard deviations and P-values (t-test, *p<0.05) are derived from n = 2 trials. C. Combined graph of rDNA signals derived from XhoI digested DNA separated by PFGE followed by Southern blotting shows that *fob1Δ mph1Δ* and *fob1Δ* have similar levels of suppression of rDNA replication in Nse5-Smc6 degron cells. Quantifications for wild-type and Nse5-Smc6 double degron cells without IAA treatment are included; representative images and FACS analyses are shown in S5 Fig.

After observing *fob1* and *mph1* suppression, we tested their genetic interactions. If Smc5/6 is required to limit Mph1 activity at Fob1-mediated fork pausing sites, we would expect combined *fob1Δ mph1Δ* to confer no additive effects on the rDNA replication phenotype of degron cells. PFGE and Southern blotting for the rDNA array showed that *fob1*Δ *mph1*Δ improved rDNA gel entry at 60, 120, and 180 min in Nse5-Smc6 double degron cells, with only a small proportion of rDNA signal remaining in the wells (Fig 5A and 5B). Quantification of several experiments shows that this improvement of rDNA replication by *fob1*Δ *mph1*Δ was not significantly greater than that shown by *fob1*Δ or *mph1*Δ single mutants (Fig 5C). In addition, the observed suppression reached a level similar to those of wild-type strains and double degron cells without IAA treatment (Fig 5C; S5 Fig). These data suggest that Mph1 and Fob1 function in the same pathways. We note that stronger effects for *fob1Δ* than *mph1Δ* at earlier time points may reflect additional roles played by Smc5/6 at rDNA beyond Mph1 regulation.

### Nse5-Smc6 double degron cells exhibit increased levels of X-mols and persistent fork pausing at rDNA

**Fig 6:**
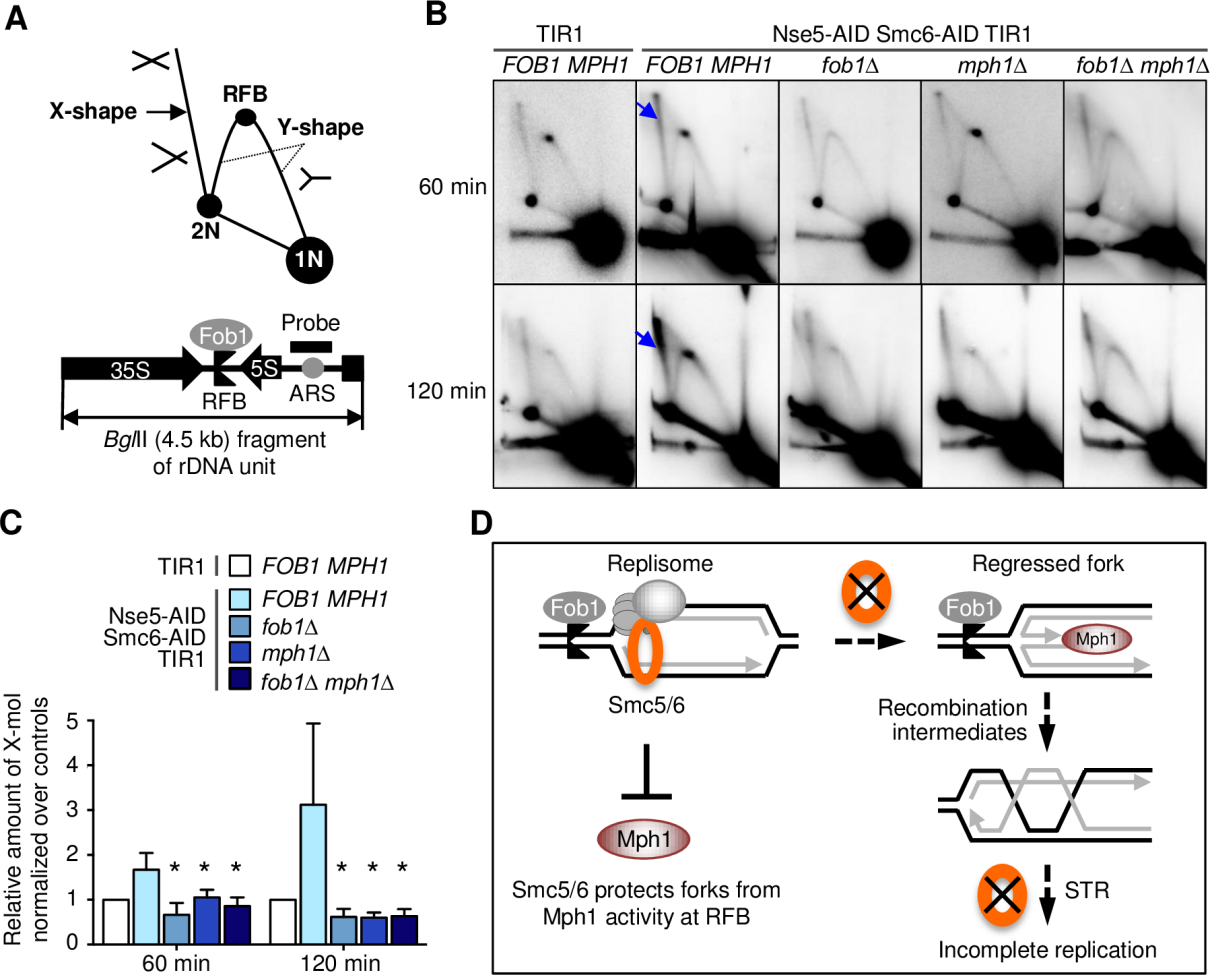
*fob1Δ* and *mph1Δ* reduce recombination structures at rDNA in Nse5-Smc6 degron cells. A. Schematic of DNA structures detected by 2D gel for the indicated BglII fragment of rDNA repeat. Top: 1N and 2N dots indicate linear DNA. Y-arc represents fork. Replication fork arrested on RFB is shown as a dot on Y-arc. X-shape structures (or X-mol) represent recombination structures (arrow). Bottom: the BglII fragment and the probe for Southern blot are shown. B. Cells from the indicated strains were collected at 60 min and 120 min after G1 release, following the scheme shown in Fig 2A. Genomic DNA was isolated, digested with BglII, and analyzed on 2D gel followed by Southern blotting with a probe depicted in (A). Arrows indicate the X-mol DNA that show an increase in degron cells compared with control cells. C. Quantification of X-mol structures at 60 min and 120 min after G1 release. Signals in the control cells were standardized to 1, and the fold-differences of other strains with respect to the control are shown. Mean and standard deviation are derived from n = 3 trials. Statistical differences between the values of degron cells and those containing *fob1Δ*, *mph1Δ*, and *fob1Δmph1Δ* were calculated by Student’s t-test (*p<0.05). D. A model of Smc5/6 function at rDNA. Smc5/6, known to be localized to rDNA, can antagonize Mph1-mediated recombination. Without Smc5/6, paused forks can engage in Mph1-mediated fork regression and subsequent recombination. As Smc5/6 is also required for supporting the STR complex in dissolving recombination intermediates, when Smc5/6 is removed, recombination intermediates can accumulate thus impeding replication completion and subsequent DNA segregation.

Our data so far support a premise that fork stalling by Fob1-RFBs in rDNA necessitates the presence of Smc5/6 to inhibit the pro-recombinogenic Mph1 activity; as such, removing Fob1 or Mph1 bypasses the need for Smc5/6. Such a potential role for Smc5/6 would mitigate recombination at RFBs and favor fork merging, an outcome less likely to cause rDNA repeat instability [29]. To test the above idea, we used 2D gel analyses to examine recombination structures formed at regions around RFB sites. An rDNA repeat fragment released by BglII restriction digest contains the RFB, rDNA replication origin (rARS), 5S rRNA gene, and part of the 35S rRNA gene (Fig 6A). As shown in previous studies, examining this fragment by 2D gel enables one to monitor levels of stalled forks at RFBs, regular replication forks (Y-shaped DNA), and recombination intermediates (X-shaped DNA or X-mols) [35] (Fig 6A).

Using the same experimental schemes as for the PFGE experiments (Fig 2A; S6A Fig), we first compared Nse5-Smc6 double degron cells and control cells with TIR1 alone. We examined samples from one S-phase time point (60 min) and one G2-phase time point (120 min) since double degron cells show reduced rDNA replication at both time points (Fig 2B–2E). Nse5-Smc6 double degron and control cells differed significantly in their X-mol or recombination intermediate levels at both time points (Fig 6B, arrows). Quantification of several experiments showed a ~1.5-fold increase at 60 min and 3-fold increase at 120 min for degron cells over controls (Fig 6C). A ~3-fold increase of RFB signals in degron cells at 120 min was also seen (Fig 6B; S6B Fig). These data suggest that the rDNA replication defect caused by Smc5/6 loss is associated with increased recombination and prolonged fork pausing.

### Fob1 and Mph1 removal reduce rDNA X-mols in Nse5-Smc6 double degron cells

We went on to ask whether increased recombination at RFB regions upon Smc5/6 loss is mediated by fork pausing and Mph1. 2D gel analyses found lower levels of recombination intermediates in both *fob1Δ* double degron and *mph1Δ* double degron strains relative to degron strains at 60 min and 120 min after G1 release; this reduction resulted in levels comparable to those of control cells (Fig 6B and 6C). As expected, *fob1Δ* also eliminated RFB-dependent fork pausing, while *mph1Δ* did not exert such an effect (Fig 6B). Moreover, the reduction in recombination intermediate levels for *fob1Δ mph1Δ* double degron cells was similar to that of *fob1Δ* or *mph1Δ* single mutants (Fig 6B and 6C), a finding consistent with our PFGE findings for rDNA array replication (Fig 5C). Together, our data suggest that both Mph1 activity and Fob1-mediated fork pausing contribute to recombination structure accumulation in the absence of Smc5/6.

### The SUMO ligase activity of Smc5/6 affects Chr XII recovery at a late time point

The above data do not exclude additional mechanisms by which Smc5/6 could influence rDNA metabolism. It has been shown that the SUMO ligase activity of the Smc5/6 complex subunit Mms21 affects nucleolar function but is not essential for growth [2,36,37]. We thus tested whether Mms21 SUMO ligase function is directly linked to rDNA replication. We found that a SUMO ligase mutant of Mms21 did not affect rDNA replication at early time points after release from G1 (S7 Fig). This is different from our data regarding Smc5/6 loss, but consistent with previous findings that sumoylation is not required for Mph1 regulation [19,33]. At a later time point, the *mms21* SUMO E3 mutant showed moderately reduced rDNA gel-entry, suggesting a late role for sumoylation. This observation corroborates a proposed role for the Mms21 SUMO ligase in dissolving recombination intermediates that block replication completion [38,39].

## Discussion

Despite being required for viability in multiple organisms, the role(s) played by Smc5/6 during mitotic growth remain poorly understood [1–3,40]. The varied phenotypes of chronic *smc5/6* mutants have complicated the delineation of specific Smc5/6 functions. Acute Smc5/6 depletion offers a strategy for bypassing the obscuring secondary effects of chronic Smc5/6 loss. An inducible protein degradation system enabled us to investigate the effects of Smc5/6 loss on DNA replication and cell cycle progression within the first cell cycle after depletion (Fig 1C–1E). This system developed for analysis of the immediate effects of robust Smc5/6 loss in yeast can stimulate the development of similar approaches in other organisms.

Combining our acute Smc5/6 depletion system with chromosomal PFGE analyses, we show that Smc5/6 complex removal causes a striking Chr XII-specific replication defect (Fig 2B–2E). This is not associated with detectable changes in cell cycle progression or markers of DNA damage and checkpoint activation, suggesting that we have isolated a primary defect of Smc5/6 loss (Fig 1D and 1E). We further show that the observed replication defect is largely localized to the rDNA array on Chr XII (Fig 3D and 3E). Our findings are in line with previous reports for *smc6-9*, but not with the chromosome size-based model derived from studies of *smc6-56* [5,6]. Considering that *smc6-56* cells experience chronic genome stress based on their altered growth and dNTP levels, adaptive responses in these cells may contribute to their overall phenotype (S2A Fig). Alternatively *smc6-56*, but not *smc6-9*, may alter the Smc5/6 function in dealing with longer chromosomes. Our finding that the Smc5/6-dependent Chr XII replication defect begins in S phase (Fig 2E) suggests that Smc5/6 plays a role in this cell cycle phase. This conclusion corroborates chromatin immunoprecipitation data localizing Smc5/6 to replication forks [41,42]. It can also be reconciled with the ability of Smc5/6 expressed from a G2-cyclin module to sustain viability, since our data suggests that even low level expression (in S phase) may be functionally adequate (Fig 1A and 1B). Taking into consideration these findings, we conclude that Smc5/6 is required beginning in S phase, particularly for rDNA replication, through to post-replicative G2 events. Our phenotypic assessment of acute Smc5/6 loss clarifies the essential function of Smc5/6 in mitotic cells and enables more reliable interpretation of *smc5/6* defects.

After redirecting focus towards an essential role of Smc5/6 in replicating at-risk rDNA loci, we provided genetic, PFGE, and 2D gel data to derive a mechanism by which Smc5/6 promotes rDNA replication. We show that Smc5/6 loss leads to increased levels of recombination intermediates at programmed rDNA fork pausing sites (Fig 6B and 6C). Importantly, removing the rDNA fork blocking protein Fob1 or the DNA helicase Mph1 in Smc5/6 degron cells reduces these intermediates and rDNA replication defects (Figs 3D, 3E, 4B, 4C, 6B and 6C). As *fob1Δ* and *mph1Δ* show similar and non-additive effects, the simplest interpretation is that upon fork stalling at RFBs in rDNA, Mph1-mediated recombination is a major contributor to defective rDNA replication when Smc5/6 is absent (Figs 5B, 5C, 6B and 6C). On the basis of these data, we propose a model in which Smc5/6 helps to manage replication forks paused at RFBs by inhibiting Mph1-mediated recombination (Fig 6D). As such, Smc5/6 is an important factor that influences the fates of stalled forks at this locus. When Smc5/6 is present, Fob1 at RFBs can prevent transcription-replication conflicts, and fork merging is favored. When Smc5/6 is absent, paused forks are vulnerable to recombination. The recombination intermediates thus generated are especially toxic, because Smc5/6 SUMOylation function is required for their dissolution [38,39]. This is consistent with our observation that *mms21* SUMO ligase mutants affect rDNA replication at a later time point (S7 Fig). This model provides a straightforward explanation for *fob1* and *mph1* suppression, as the former reduces the number of forks that require Smc5/6 protection from Mph1 activity, while the latter reduces the potential for paused forks to undergo recombination. Both genetic changes decrease the Smc5/6 requirement at rDNA. As the *fob1* and *mph1* suppression patterns are not entirely identical, they must also play unique, yet-to-be determined roles in mediating Smc5/6 effects on rDNA replication.

Our data also reveal a previously unappreciated function of Mph1 at the rDNA locus. Although recombination at RFBs is toxic when Smc5/6 is lost, such repair could be useful for adjusting rDNA repeat numbers. Whether and how cells enable restricted use of Mph1 for this purpose will be interesting to investigate in the future. Mph1 and its homologs have been suggested to promote recombination at stalled replication forks via their ability to regress forks, which entails the annealing of two nascent strands accompanied by re-annealing of their template strands [32]. Replication fork regression can provide a mechanism for replication restart, but also generate DNA structures prone to cleavage or recombination [32]. As such, fork regression is negatively regulated by multiple mechanisms; Smc5/6 participates in one such mechanism by directly binding to and preventing Mph1 oligomerization at fork junctions [32, 33]. Though fork regression is mostly investigated in DNA damage conditions, our findings suggest that it can also occur in situations of endogenous fork pausing. In conjunction with previous findings of the Smc5/6-Mph1 relationship under DNA damage conditions, our data helps to establish the concept that a key Smc5/6 function is regulation of Mph1 in multiple conditions.

Overall, our results suggest that Smc5/6 plays a primary role in managing programmed fork pausing at rDNA by inhibiting pro-recombinogenic Mph1 activity. We find this role to be rDNA-specific, as other chromosomes harboring protein barriers [43,44] replicate proficiently upon Smc5/6 depletion and artificially introduced protein barriers on Chr III did not affect its replication in double degron cells [31] (S4 Fig). We suggest that multiple features unique to rDNA may help explain why Smc5/6 is particularly indispensable at this locus. First, the presence of many RFB sites in rDNA can generate much higher levels of replication fork pausing than any other locus. Second, some proteins that facilitate replication are known to be excluded from the nucleolus [45]. As such, Smc5/6, known to be enriched at rDNA [7], could be particularly important for managing stalled forks at this locus. When forks are paused outside rDNA, other pathways may compensate for the lack of Smc5/6 activity, so they do not manifest defects as robustly upon Smc5/6 loss. Third, rDNA has a unique chromatin environment and architecture connected to its activities in transcription, ribosome assembly, and mitotic exit. This high level of DNA, RNA, and protein transactions may generate a specific demand for Smc5/6 function. Finally, rDNA replication continues at the end of S and into G2 phase when other chromosomes have completed their replication [29]. This could further generate a demand for Smc5/6. In-depth examination of which and how these unique rDNA features influence its duplication and pose a critical requirement for Smc5/6 will provide additional insight into how this highly conserved repetitive sequence sustains stability across evolution.

In human cells, Smc5/6 also promotes replication and has been shown to localize to a subset of stalled replication forks [46,47]. Thus, our work also stimulates the examination of a similar role for human Smc5/6 in managing fork pausing in rDNA and other repetitive or highly organized regions where alternative mechanisms of fork management are ineffective. It is worth noting that disrupting rDNA replication has far-reaching consequences beyond this locus, through its strong influences on mitotic exit, nuclear organization, transcription, and translation [48,49]. Indeed, yeast Smc5/6 mutants show nucleolar fragmentation and stress [2,37]. As such, the importance of Smc5/6 function to rDNA replication may help explain the divergent phenotypes underlying its two human syndromes, which feature pleiotropic developmental defects across multiple lineages, including the musculoskeletal, endocrine and immune systems [4,50].

## Methods

### Yeast strains and general manipulation

Yeast strains are derivatives of W1588-4C, a *RAD5* variant of W303 (*MAT*a *ade2-1 can1-100 ura3-1 his3-11*,*15 leu2-3*,*112 trp1-1 rad5-535*) [2]. At least two strains per genotype were examined in each experiment, and only strain is listed for each genotype in S1 Table. Standard methods were used for yeast strain construction. To tag subunits of the Smc5/6 complex, PCR products containing AID and FLAG tag sequences were generated with flanking homologous sequences to the insertion site. Standard PCR integration methods were used to generate fusion constructs, which were then fully sequenced to confirm the correct tagging.

### Cell cycle arrest and release

Two main protocols were used for cell cycle studies, as shown in Figs 1D and 2A. In Fig 1D, asynchronous cultures were arrested in G1 phase by adding 5μg/ml α-factor (ThermoFisher) to media for 90 min. 1 mM IAA (Sigma) was added for 90 min to degrade Nse5-AID and Smc6-AID proteins. Subsequently, cells were released into fresh YPD containing 1 mM IAA to sustain Nse5 and Smc6 depletion in degron cells. Samples were collected at several time points after release as indicated. Note that the same procedure was applied to cells without degron alleles to ensure parallel treatment. A similar experimental procedure shown in Fig 2A has two alterations. One is BrdU (Sigma) addition after cells were released from G1 arrest to monitor new DNA synthesis. Another is nocodazole (US Biologicals) addition to cultures 45 min after G1 release to prevent first cell cycle exit. Standard FACS analyses were performed as described previously [51].

### 2D agarose gel electrophoresis

2D gel analyses were performed as previously described [52]. DNA was extracted and digested by BglII and separated by agarose gel electrophoresis in two dimensions. DNA was transferred onto Hybond-XL membranes (GE Healthcare) and analyzed by Southern blot using probes hybridizing specifically to rDNA. Primers used for probe amplification are available upon request. For quantification, the signals of 1N DNA were obtained from shorter exposures, while those of DNA intermediates came from longer exposures to ensure both types of signals fell within linear range of detection on the PhosphorImager.

### PFGE analysis, BrdU blotting, and Southern blots

PFGE was performed as previously described [53]. In brief, cells harvested from the indicated time points were embedded in agarose plugs, spheroplasted, and deproteinized. Plugs were loaded into 0.5X TBE gels and run on a Bio-Rad CHEF-DR III Pulsed Field Electrophoresis System for 12 hours to achieve chromosome separation. Gels were stained by ethidium bromide and Sytox (Molecular Probes) and then transferred onto Hybond-N+ membranes (GE Healthcare) using standard capillary transfer technique. Membranes were probed with anti-BrdU antibody (BD) and α-mouse secondary antibody (GE Healthcare). Membranes were scanned with Fujifilm LAS-3000 luminescent image analyzer, which has a linear dynamic range of 10^4^ to achieve reliable quantification. The percentage of gel entry for each chromosome was calculated by dividing the chromosomal band signal by the sum of chromosomal band signal and well signal, after background subtraction. The positions of each chromosome were derived from [54]. Southern blotting of Chr XII, Chr III, and rDNA were performed using specific probes hybridizing to each region, and primers used for probe amplification are available upon request.

### Other methods

To detect protein levels, standard TCA protein extraction methods were used [55]. Protein samples were resolved on 3–8% or 4–20% gradient gels (Life Technologies and Bio-Rad) and transferred onto 0.2 um nitrocellulose membranes (G5678144, GE). Antibodies used were: α-Myc (9E10, Bio X Cell), α-HA (F-7, Santa Cruz), α-Flag M2 (Sigma), α-V5 (Life Technologies), α-Clb2 (y-180, Santa Cruz), α-Pds1 (gift of E. Schiebel), α-Rad53 (yC-19, Santa Cruz), α-H2A pS129 (Abcam), and PAP (P1291, Sigma). dNTP quantification was performed as previously described [56].

## Supporting information

S1 Fig The AID degron system and degradation of Smc5/6 subunits. **A**. Schematics to highlight the AID degron approach. TIR1 is a plant ubiquitin (Ub) E3 adapter protein that can associate with the yeast SCF Ub E3 ligase complex via its cullin subunit. The target protein fused to a plant AID module can associate with TIR1 with IAA as a bridge molecule. IAA thus induces proximity between the AID target protein fusion and the Ub ligase, leading to ubiquitination and proteomic degradation of the former.
(**B-D)** AID-mediated degradation of Smc5/6 subunits generally does not affect the stability of the other substrates of the complex. A few examples of proteins blots are shown where degradation of the indicated AID-tagged subunit leaves unchanged the protein levels of other tagged subunits in the complex. Protein levels before IAA addition and after 90 mins of IAA treatment are shown.
(TIF)

S2 Fig dNTP levels in hypomorphic Smc5/6 mutants and the Nse5-Smc6 double degron cells. **A**. dNTP pools were measured for wild-type (WT), *mms21-11*, *smc6-P4*, and *smc6-56* cells. Mean and standard deviations are derived from n = 2 trials; P-values are shown for values between mutants and wild-type cells (t-test, *p<0.05, **p<0.01).
**B**. dNTP pools were measured for wild-type (WT), strains containing TIR1 alone, and the Nse5-Smc6 double degron cells. In each case, both asynchronous and G1-arrested cells were examined. Mean and standard deviations are derived from n = 2 trials; the values between wild-type and TIR1 alone cells are not statistic different, as those between TIR alone and double degron cells (student t-test).
(TIF)

S3 Fig Nse5-Smc6 double degron cells are defective in replicating Chr XII but not other chromosomes. A. PFGE gels shown in Fig 2C was examined by staining with EtBr and Sytox.
B. Quantification of signals for each BrdU-labeled chromosome band was normalized to the total DNA stain signal in each lane. The BrdU signal of all chromosomes except Chr XII were calculated as a sum (“All Other Chromosomes”). All values were normalized using the highest Control value as 1. Standard deviations and P-values (t-test, *p < 0.05, **p < 0.01) are derived from n = 3 trials.
C. PFGE gels shown in Fig 2E was examined by staining by ethidium bromide and Sytox.
(TIF)

S4 Fig Smc5/6 loss does not affect replication of Chr III harboring RFB sites. A. Diagram depicts the Chr III harboring two RFB sites that have been shown to temporally pause replication forks emanated from two nearby origins (ARS305 and ARS306) upon Fob1 over expression driven by galactose inducible promoter. Restriction enzyme sites and the probe used for 2D gel analysis in panel E are indicted.
B. Experimental scheme to induce Fob1 expression and Smc5/6 degradation before cells entering S phase and examination of multiple time points in S and G2/M phases.
C. PFGE gels stain to show that Smc5/6 loss reduces the replication of Chr XII but not Chr III that harbors RFB sites upon Fob1 overexpression. Double degron cells containing Gal-Fob1 and Chr III-RFB PFGE to visualize replication completion.
D. FACS analyses of samples in panel **C**. Note that cell cycle progression in galactose media is slower than those in glucose media in other figures.
E. 2D gel analysis confirms replication fork pausing at the RFB site near ARS306 upon Fob1 over-expression. Samples collected as in panel C and D (+Galactose) and in control conditions without Fob1 overexpression (+Raffinose) were subjected 2D gel analyses. The *Sal*I fragments indicted in panel A was examined using a probe near RFB (grey bar in A). Signals of paused replication forks in this fragment (black arrow) were observed in three S phase time points only in Galactose conditions.
F. FACS analyses of samples in panel **E.**
(TIF)

S5 Fig PFGE examination of rDNA array replication of wild-type and Nse5-Smc6 double degron cells without IAA treatment. A. FACS profile showing cell cycle progression of indicated cells without IAA treatment. Experiments followed the schema shown in Fig 2A.
B. XhoI-digested samples were subjected to PFGE and Southern blot to examine rDNA array replication, as described in Fig 4A.
(TIF)

S6 Fig A. FACS analyses of samples shown in Fig 6B.
B. Quantification of the relative RFB levels in the Nse5-Smc6 double degron cells over those in the control TIR1 cells. Mean and standard deviation are derived from n = 3 trials. The statistic differences between the values of degron cells and controls were calculated by student t-test (*p<0.05).
(TIF)

S7 Fig PFGE examination of rDNA array of wild-type and mms21-CH cells. A. FACS profile showing cell cycle progression of wild-type cells and mms21-CH, a SUMO E3 mutant. Experiments followed the schema shown in Fig 2A.
B. XhoI-digested samples were subjected to PFGE and Southern blot to examine rDNA array replication. Signals from an rDNA-specific probe are quantified as in Fig 4A. Standard deviations and P-values (t-test, *p<0.05) are derived from n = 2 trials.
(TIF)

S1 Table Strains used in the study. (DOCX)
