# Supplementary figures and images for: Acute Smc5/6 depletion reveals its primary role in rDNA replication by restraining recombination at fork pausing sites
Source: PLoS Genet. 2018 Jan 23;14(1):e1007129. doi: 10.1371/journal.pgen.1007129 (PMC5779651; doi:10.1371/journal.pgen.1007129)

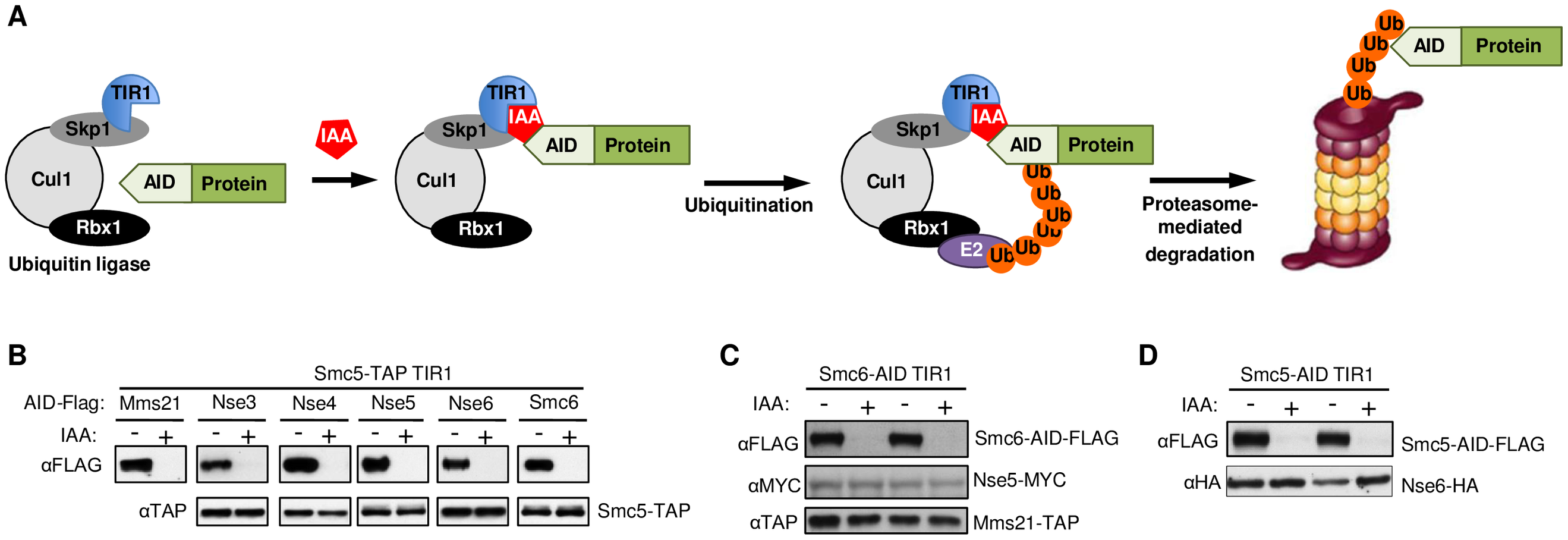

Supplement: S1 Fig — A. Schematics to highlight the AID degron approach. TIR1 is a plant ubiquitin (Ub) E3 adapter protein that can associate with the yeast SCF Ub E3 ligase complex via its cullin subunit. The target protein fused to a plant AID module can associate with TIR1 with IAA as a bridge molecule. IAA thus induces proximity between the AID target protein fusion and the Ub ligase, leading to ubiquitination and proteomic degradation of the former. (B-D) AID-mediated degradation of Smc5/6 subunits generally does not affect the stability of the other substrates of the complex. A few examples of proteins blots are shown where degradation of the indicated AID-tagged subunit leaves unchanged the protein levels of other tagged subunits in the complex. Protein levels before IAA addition and after 90 mins of IAA treatment are shown. (TIF) [file pgen.1007129.s001.tif]

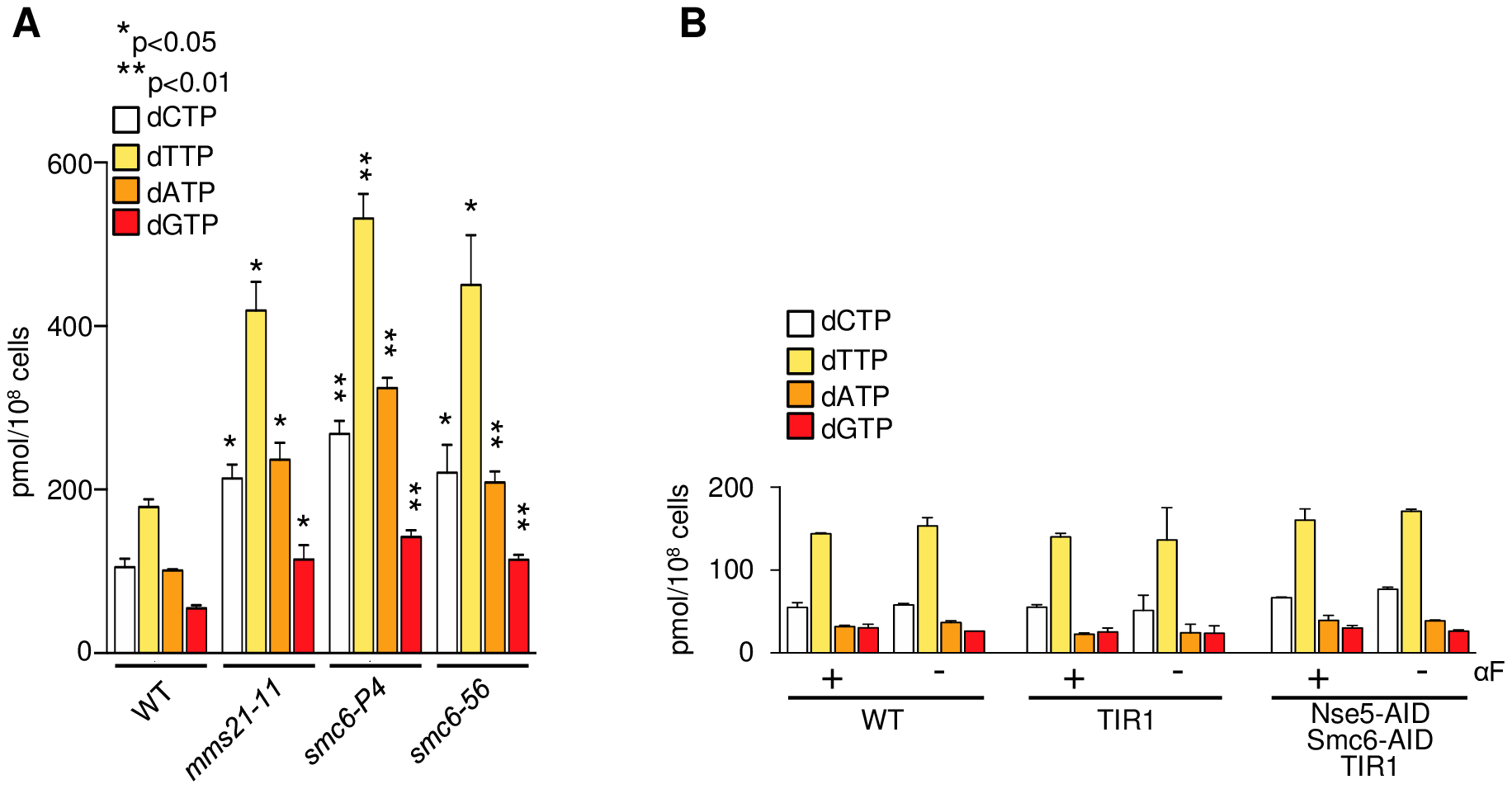

Supplement: S2 Fig — A. dNTP pools were measured for wild-type (WT), mms21-11, smc6-P4, and smc6-56 cells. Mean and standard deviations are derived from n = 2 trials; P-values are shown for values between mutants and wild-type cells (t-test, *p<0.05, **p<0.01). B. dNTP pools were measured for wild-type (WT), strains containing TIR1 alone, and the Nse5-Smc6 double degron cells. In each case, both asynchronous and G1-arrested cells were examined. Mean and standard deviations are derived from n = 2 trials; the values between wild-type and TIR1 alone cells are not statistic different, as those between TIR alone and double degron cells (student t-test). (TIF) [file pgen.1007129.s002.tif]

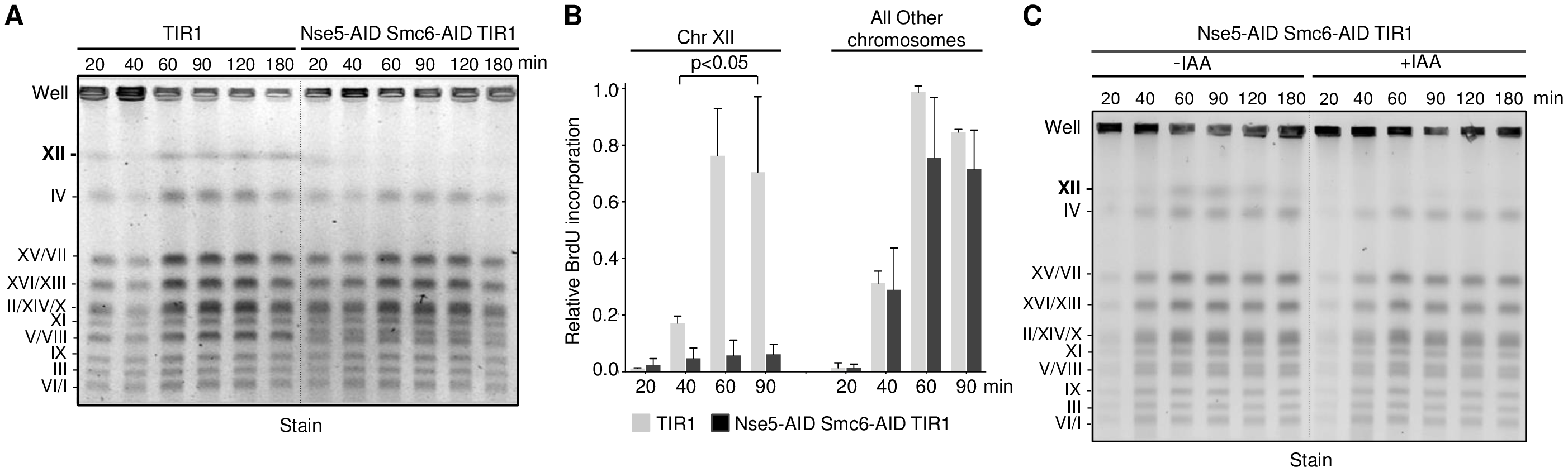

Supplement: S3 Fig — A. PFGE gels shown in Fig 2C was examined by staining with EtBr and Sytox. B. Quantification of signals for each BrdU-labeled chromosome band was normalized to the total DNA stain signal in each lane. The BrdU signal of all chromosomes except Chr XII were calculated as a sum (“All Other Chromosomes”). All values were normalized using the highest Control value as 1. Standard deviations and P-values (t-test, *p < 0.05, **p < 0.01) are derived from n = 3 trials. C. PFGE gels shown in Fig 2E was examined by staining by ethidium bromide and Sytox. (TIF) [file pgen.1007129.s003.tif]

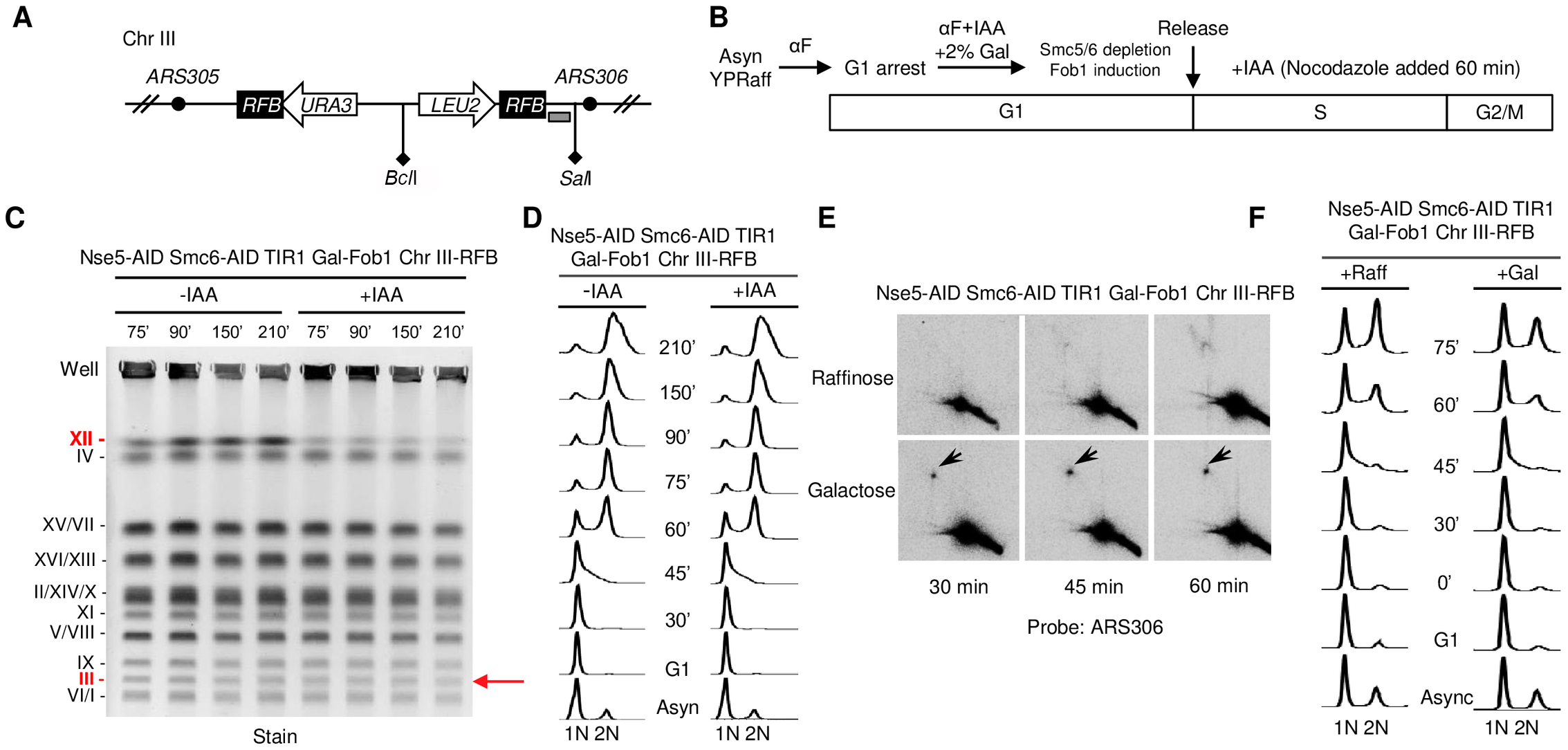

Supplement: S4 Fig — A. Diagram depicts the Chr III harboring two RFB sites that have been shown to temporally pause replication forks emanated from two nearby origins (ARS305 and ARS306) upon Fob1 over expression driven by galactose inducible promoter. Restriction enzyme sites and the probe used for 2D gel analysis in panel E are indicted. B. Experimental scheme to induce Fob1 expression and Smc5/6 degradation before cells entering S phase and examination of multiple time points in S and G2/M phases. C. PFGE gels stain to show that Smc5/6 loss reduces the replication of Chr XII but not Chr III that harbors RFB sites upon Fob1 overexpression. Double degron cells containing Gal-Fob1 and Chr III-RFB PFGE to visualize replication completion. D. FACS analyses of samples in panel C. Note that cell cycle progression in galactose media is slower than those in glucose media in other figures. E. 2D gel analysis confirms replication fork pausing at the RFB site near ARS306 upon Fob1 over-expression. Samples collected as in panel C and D (+Galactose) and in control conditions without Fob1 overexpression (+Raffinose) were subjected 2D gel analyses. The SalI fragments indicted in panel A was examined using a probe near RFB (grey bar in A). Signals of paused replication forks in this fragment (black arrow) were observed in three S phase time points only in Galactose conditions. F. FACS analyses of samples in panel E. (TIF) [file pgen.1007129.s004.tif]

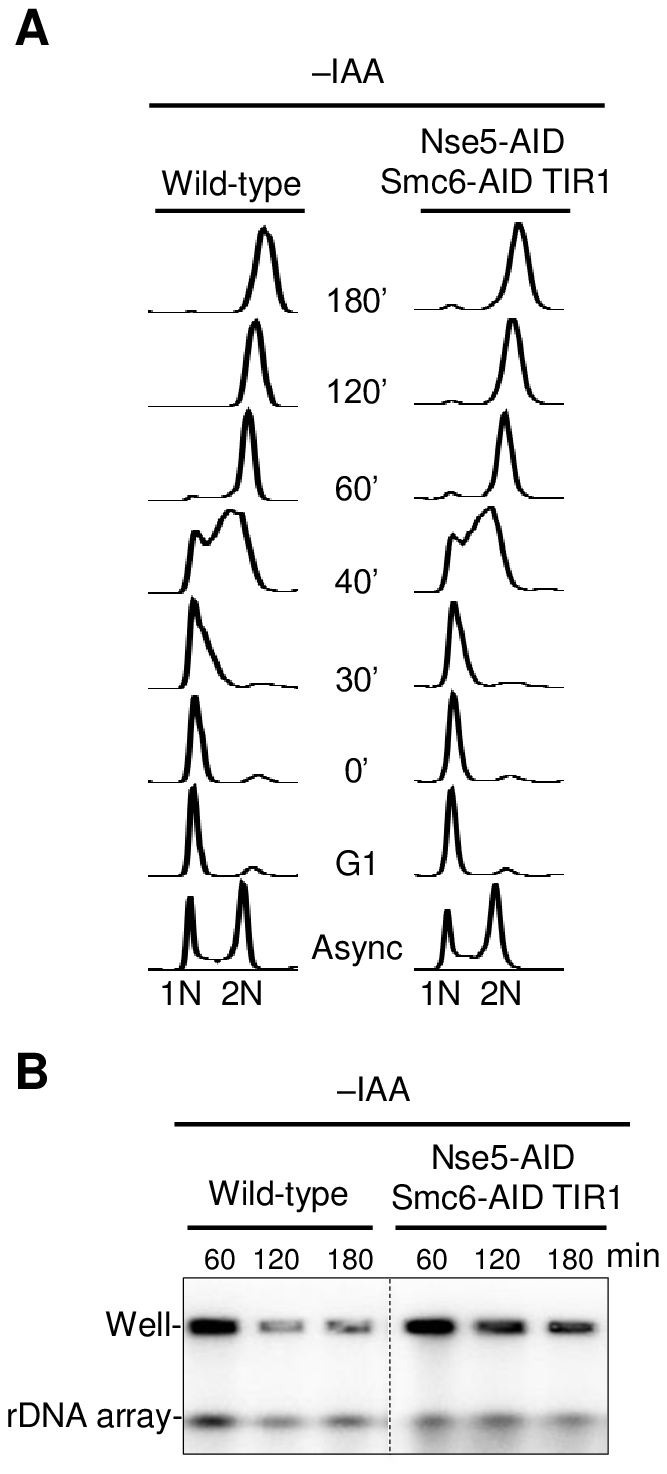

Supplement: S5 Fig — A. FACS profile showing cell cycle progression of indicated cells without IAA treatment. Experiments followed the schema shown in Fig 2A. B. XhoI-digested samples were subjected to PFGE and Southern blot to examine rDNA array replication, as described in Fig 4A. (TIF) [file pgen.1007129.s005.tif]

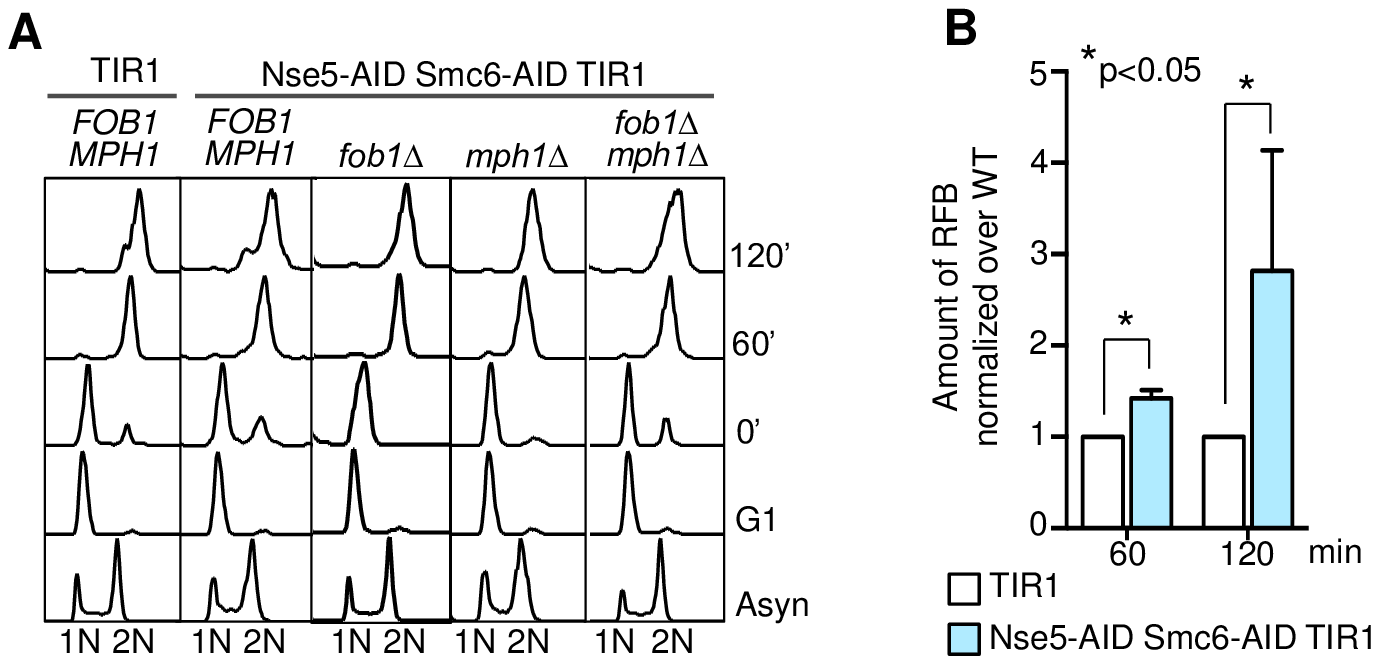

Supplement: S6 Fig — A. FACS analyses of samples shown in Fig 6B. B. Quantification of the relative RFB levels in the Nse5-Smc6 double degron cells over those in the control TIR1 cells. Mean and standard deviation are derived from n = 3 trials. The statistic differences between the values of degron cells and controls were calculated by student t-test (*p<0.05). (TIF) [file pgen.1007129.s006.tif]

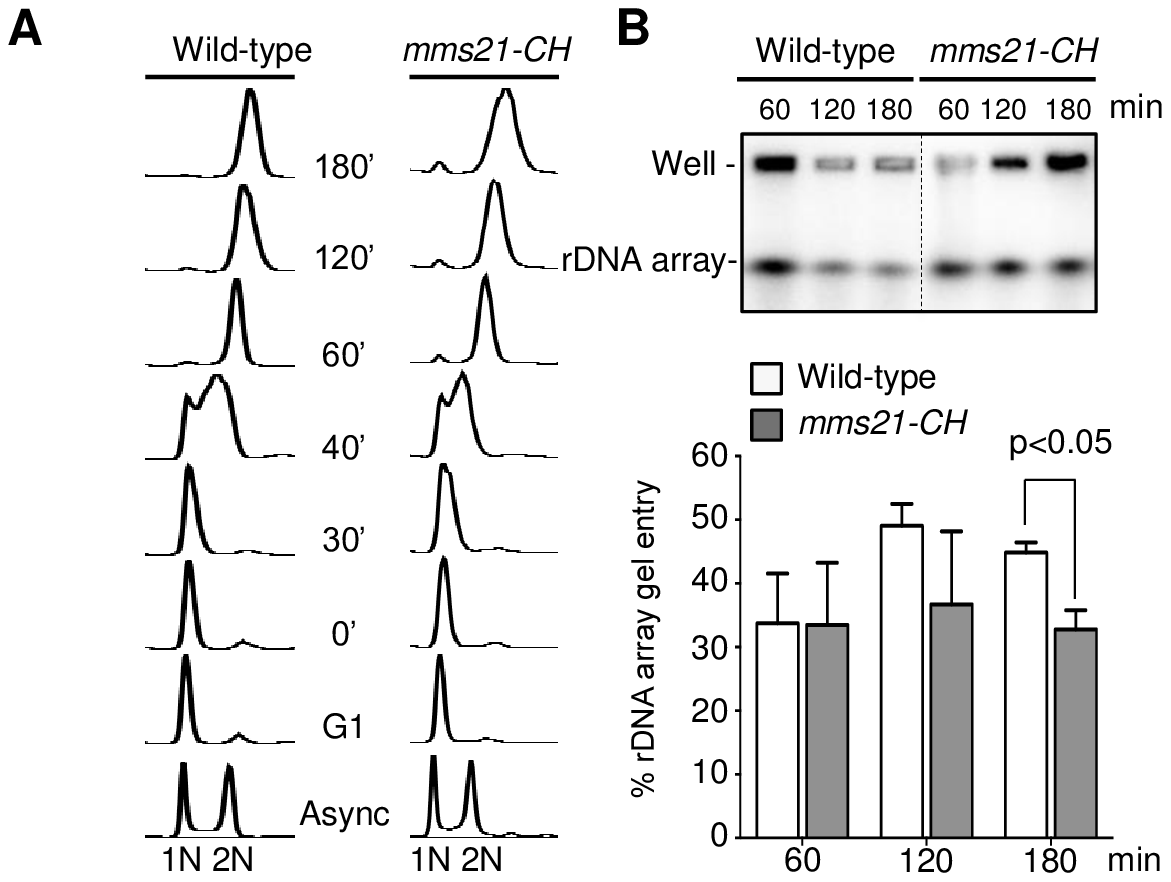

Supplement: S7 Fig — A. FACS profile showing cell cycle progression of wild-type cells and mms21-CH, a SUMO E3 mutant. Experiments followed the schema shown in Fig 2A. B. XhoI-digested samples were subjected to PFGE and Southern blot to examine rDNA array replication. Signals from an rDNA-specific probe are quantified as in Fig 4A. Standard deviations and P-values (t-test, *p<0.05) are derived from n = 2 trials. (TIF) [file pgen.1007129.s007.tif]
