# Supplementary material for: Acute Smc5/6 depletion reveals its primary role in rDNA replication by restraining recombination at fork pausing sites
Source: PLoS Genet. 2018 Jan 23;14(1):e1007129. doi: 10.1371/journal.pgen.1007129 (PMC5779651; doi:10.1371/journal.pgen.1007129)
Supplement: S1 Table — (DOCX) [file pgen.1007129.s008.docx]

**S1 Table. Strains used in the study**

| **Strain Name** | **Genotype** |
| --- | --- |
| T1130-6A | W303 *MAT*a *ade2-1 can1-100 ura3-1 his3-11,15 leu2-3,112 trp1-1 RAD5* |
| X136-13D | *mms21-11::LEU2* |
| T605-14B | *smc6-P4-13MYC::KANMX* |
| T606-7A | *smc6-56-13MYC::KANMX* |
| T1130-6C | *ADH1::OsTIR1-9MYC::URA3* |
| T1107-5B | *SMC5-AID-3FLAG::KANMX ura3-1::ADH1-OsTIR1-9MYC::URA3* |
| T1107-5D | *SMC5-AID-3FLAG::KANMX* |
| T1105-7B | *SMC6-AID-3FLAG::KANMX ura3-1::ADH1-OsTIR1-9MYC::URA3* |
| T1105-7D | *SMC6-AID-3FLAG::KANMX* |
| T1128-4A | *MMS21-AID-3FLAG::HIS3 ura3-1::ADH1-OsTIR1-9MYC::URA3* |
| T1128-2A | *MMS21-AID-3FLAG::HIS3* |
| T1129-5B | *NSE3-AID-3FLAG::HIS3 ura3-1::ADH1-OsTIR1-9MYC::URA3* |
| T1129-3B | *NSE3-AID-3FLAG::HIS3* |
| T1130-6D | *NSE4-AID-3FLAG::KANMX ura3-1::ADH1-OsTIR1-9MYC::URA3* |
| T1130-6B | *NSE4-AID-3FLAG::KANMX* |
| T1110-4A | *NSE5-AID-FLAG::HIS3 ura3-1::ADH1-OsTIR1-9MYC::URA3* |
| T1110-4C | *NSE5-AID-FLAG::HIS3* |
| T1134-7B | *NSE6-AID-FLAG::KANMX ura3-1::ADH1-OsTIR1-9MYC::URA3* |
| T1134-7A | *NSE6-AID-FLAG::KANMX* |
| X4715-11A | *SMC6-AID-FLAG::HIS3 ura3-1::ADH1-OsTIR1-9MYC::URA3 MMS21-TAP::TRP1* |
| X4580-9B | *SMC6-AID-3FLAG::KANMX ura3-1::ADH1-OsTIR1-9MYC::URA3 NSE5-13MYC::TRP* |
| X4410-17A | *SMC6-AID-3FLAG::KANMX ura3-1::ADH1-OsTIR1-9MYC::URA3 SMC5-TAP::KAN MMS21-3HA::TRP* |
| X4619-4C | *SMC5-AID-3FLAG::KANMX ura3-1::ADH1-OsTIR1-9MYC::URA3 NSE6-3HA::HIS RTT107-13MYC::KAN* |
| X4458-15A | *NSE3-AID-3FLAG::HIS3 ura3-1::ADH1-OsTIR1-9MYC::URA3 SMC5-TAF::KAN* |
| X4413-11A | *NSE4-AID-3FLAG::KANMX ura3-1::ADH1-OsTIR1-9MYC::URA3 SMC5-TAP::KAN MMS21-3HA::TRP* |
| X4460-10C | *NSE5-AID::KANMX ura3-1::ADH1-OsTIR1-9MYC::URA3 SMC5-TAF::KAN* |
| X4461-6B | *NSE6-AID-FLAG::HIS3 ura3-1::ADH1-OsTIR1-9MYC::URA3 SMC5-TAF::KAN* |
| X5431-14D | *SMC6-AID-3FLAG::HIS3 NSE5-AID::KANMX ura3-1::ADH1-OsTIR1-9MYC::URA3 GPD1::OsTIR1::LEU2* |
| X6923-2-10C | *SMC6-AID-3FLAG::HIS3 NSE5-AID::KANMX ADH1::OsTIR1-9MYC::TRP1 GPD1::OsTIR1::LEU2 URA3::GPD-TK7* |
| X6412-14C | *ADH1::OsTIR1-9MYC::TRP1 GPD1::OsTIR1::LEU2 URA3::GPD-TK7* |
| X6740-5-17D | *SMC6-AID-3FLAG::HIS3 NSE5-AID::KANMX ADH1::OsTIR1-9MYC::TRP1 GPD1::OsTIR1::LEU2 URA3::GPD-TK7 fob1Δ::HYG* |
| X6513-2-22D | *SMC6-AID-3FLAG::HIS3 NSE5-AID::KANMX ADH1::OsTIR1-9MYC::TRP1 GPD1::OsTIR1::LEU2 URA3::GPD-TK7 mph1∆::KAN* |
| X6924-3-7B | *SMC6-AID-3FLAG::HIS3 NSE5-AID::KANMX ADH1::OsTIR1-9MYC::TRP1 GPD1::OsTIR1::LEU2 URA3::GPD-TK7 mph1∆::KAN fob1Δ::HYG* |
| X3622-6D | *Mat a mms21-CH::HIS* |
| X7577-3D | *SMC6-AID-3FLAG::HIS3 NSE5-AID::KAN ADH1-OsTIR1-9MYC::URA3 ARS305-RFB-(URA3)-(LEU2)-(RFB)-ARS306 GAL-FOB1-TRP1* |
